# Pyrin- and CARD-only Proteins as Regulators of NLR Functions

**DOI:** 10.3389/fimmu.2013.00275

**Published:** 2013-09-17

**Authors:** Hongnga T. Le, Jonathan A. Harton

**Affiliations:** ^1^Center for Immunology and Microbial Disease, Albany Medical College, Albany, NY, USA; ^2^Department of Biochemistry, Faculty of Biology, University of Science, Vietnam National University-Ho Chi Minh City, Ho Chi Minh City, Vietnam

**Keywords:** pyrin, CARD, NLR, inflammasome, NF-kappaB, inhibitors

## Abstract

Upon activation Nod-like receptors (NLRs) assemble into multi-protein complexes such as the NODosome and inflammasome. This process relies upon homo domain interactions between the structurally related Pyrin and caspase-recruitment (CARD) domains and adaptor proteins, such as ASC, or effector proteins, such as caspase-1. Although a variety of NLRP and NLRC complexes have been described along with their activating stimuli and associated proteins, less familiar are processes limiting assembly and/or promoting dissociation of NLR complexes. Given the importance of limiting harmful, chronic inflammation, such regulatory mechanisms are significant and likely numerous. Proteins comprised of a solitary Pyrin domain (Pyrin-only) or CARD domain (CARD-only) posses an obvious potential ability to act as competitive inhibitors of NLR complexes. Indeed, both Pyrin-only proteins (POPs) and CARD-only proteins (COPs) have been described as regulators of caspase-1 and/or NLR-inflammasome activation and not surprisingly as factors mediating pathogenesis. Although clear examples of pathogen encoded POPs are currently limited to members of the poxviridae, the human genome likely encodes three POPs (POP1, POP2, and a potential POP3), of which only POP2 is known to prevent NLR:ASC interaction, and three COPs (COP/Pseudo-ICE, INCA, and ICEBERG), initially described for their ability to inhibit caspase-1 activity. Surprisingly, among eukaryotic species POPs and COPs appear to be evolutionarily recent and restricted to higher primates, suggesting strong selective pressures driving their emergence. Despite the importance of understanding the regulation of NLR functions, relatively little attention has been devoted to revealing the biological impact of these intriguing proteins. This review highlights the current state of our understanding of POPs and COPs with attention to protein interaction, functions, evolution, implications for health and disease, and outstanding questions.

## Introduction

Inflammation is a non-specific, physiological response of the immune system to infection and injury. Acute inflammation occurs within a few minutes following the injury of tissues. This process is initiated by tissue-associated cells, such as resident macrophages, that release inflammatory mediators, increase permeability of the blood vessels, and subsequently recruit phagocytes to the affected sites, eliminating not only invading organisms but damaged tissues as well. Although acute inflammation is normally self-limiting and beneficial for host defense and healing, excessive inflammation or prolonged (chronic) inflammation is deleterious and a cardinal, if not causative, feature of many diseases.

Among the mediators of inflammation, IL-1β is a prominent pro-inflammatory cytokine produced primarily by myeloid cells that efficiently stimulates the expression of other gene products, such as IL-6 and acute phase proteins associated with inflammation, thus initiating a self-amplifying cytokine network (1). IL-1β is also key to the fever response and vascular changes accompanying inflammation. It is well-known that IL-1β, together with the closely related cytokine IL-18, is matured through catalytic processing by caspase-1, an event associated with the assembly of multi-protein, caspase-1 activating complexes known as inflammasomes. A closely related protein complex, the NODosome, also promotes inflammation through activation of transcription factors promoting expression of the proform of IL-1β as well as other inflammatory cytokines including TNFα and IL-6 [reviewed in (2)]. Moreover, an increasing number of publications highlight the importance of various specific inflammasome/NODosome complexes in not only normal inflammatory responses, but in human pathologies ranging from metabolic disorders to autoimmune diseases and cancer [reviewed in (3–5)].

Most inflammasomes (and all NODosomes) result from the activation of intracellular sensor proteins belonging to the nucleotide-binding, leucine-rich repeat (LRR), or NOD-like receptor family (NLR). NLR agonists include pathogen-derived molecules [e.g., lipopolysaccharide, muramyl dipeptides (MDPs), and flagellin] as well as “sterile” substances (e.g., asbestos, silica, cholesterol, or uric acid crystals) [reviewed in (6, 7)]. The non-NLR dsDNA sensor AIM2 also seeds an inflammasome complex (8). In all cases, formation of these active complexes requires homotypic domain interactions involving Pyrin domains (PYD), caspase-recruitment (CARD) domains, or both.

Given the broad significance of inflammasomes, understanding their regulation is a topic of critical importance. Beyond regulation of expression and preventing inflammasome assembly/activation, mechanisms that limit that assembly or favor disassembly of these complexes have obvious implications for controlling inflammasome-mediated inflammation. Interestingly, two groups of proteins, Pyrin-only proteins (POPs) and CARD-only proteins (COPs) have been recently described as negative modulators that impact and likely regulate inflammasome assembly and/or caspase-1 dependent production of IL-1β. NODosomes also rely upon CARD domains and their activation may also be regulated by COPs. Although not well-studied and underappreciated, these molecules are known, or strongly implied, to interfere with either inflammasome adapter molecule interaction or downstream recruitment of caspase-1. In addition, POPs and COPs, like other PYD and CARD-containing proteins, can influence the activation of NF-κB.

## Nod-Like Receptors

**Figure 1:**
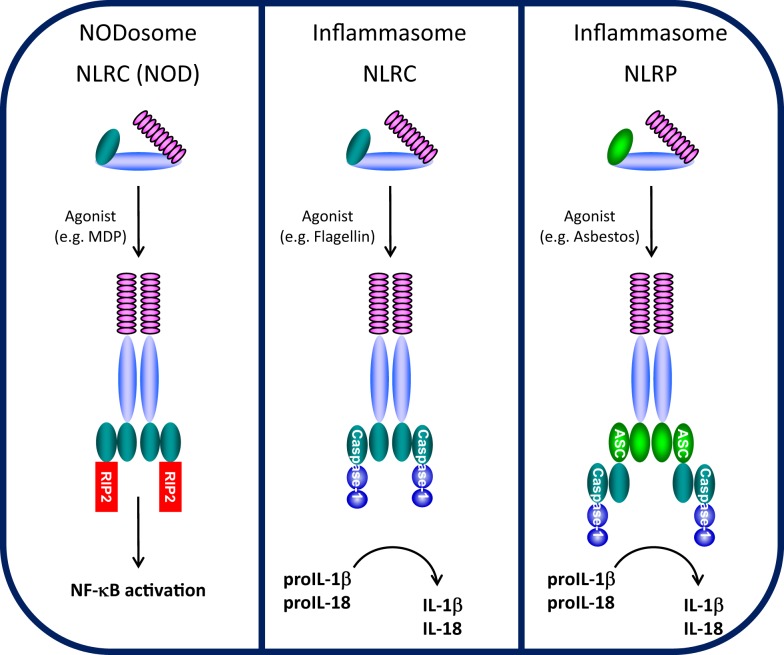
**NODosome and inflammasome complexes assembled through CARD–CARD and PYD–PYD homotypic interactions**. CARD domain-dependent direct recruitment of RIP-2 by NLRCs (e.g., NOD2/NLRC2) leads to NF-κB activation (left panel) and CARD domain-dependent direct recruitment of Caspase-1 by NLRC4 leads to IL-1β/IL-18 processing (middle panel), while PYD domain-dependent recruitment of the adaptor ASC (PYCARD) leads to CARD domain-dependent recruitment of Caspase-1 and IL-1β/IL-18 processing (right panel). (Domains: purple, LRRs; light blue, NBD/NACHT; teal, CARD; light green, PYD).

Cells are able to recognize and respond to a large array of common molecules by virtue of a set of diverse, but limited, pattern recognition receptors (PRRs). PRRs have been characterized into four groups, Toll-like receptors (TLRs), RIG-I-like receptors (RLRs), AIM2-like receptors (ALRs), and NLRs. While TLRs, a family of classical transmembrane proteins well-known as important for recognizing either extracellular or membrane-encased foreign organisms (9), form the front line of innate immune sensors, NLRs constitute a second, intracellular line. The NLR family consists of intracellular soluble proteins that sense cytosolic pathogen-associated molecular patterns (PAMPs) as well as a range of environmental- and host-derived stress signals, also known as danger-associated molecular patterns (DAMPs). Conserved tripartite-domain proteins, NLRs contain a central nucleotide-binding and oligomerization domain (NOD, NBD, NACHT), C-terminal LRR similar to those of TLRs that may serve as a “sensor domain,” and a N-terminal effector domain. NLRs are thought to be synthesized in an auto-repressed, inactive form where an intramolecular interaction between the LRR and NACHT domains is proposed to block NACHT-mediated oligomerization, thus inhibiting NLR auto-activation (10). Upon binding (or responding to) respective ligands, the LRRs are thought to undergo a conformational change allowing NACHT-dependent oligomerization and recruitment of appropriate adaptor proteins, leading to NODosome or inflammasome assembly (Figure 1).

### NODosomes

The NLRC (NOD) and NLRP (NALP) subfamilies which contain a CARD or PYD N-terminal effector domain, respectively, are the most studied NLRs (11). NOD1/NLRC1 (CARD 4) and NOD2/NLRC2 (CARD 15) were among the first NLRs to be described (12, 13). NOD1 and NOD2 both detect muropeptides released from bacterial peptidoglycan (14). NOD1 binds to diaminopimelic acid (DAP) (strictly in Gram-negative bacteria), whereas NOD2 directly interacts to MDP (found in both Gram-positive and Gram-negative bacteria) (15–18). Upon specific ligand recognition, NACHT domain oligomerization initiates recruitment of the CARD-containing kinase RIP-2 through a homotypic CARD–CARD interaction, leading to the formation of NODosomes (Figure 1). RIP-2, in turn, activates the IκB kinase (IKK) complex followed by the subsequent release and nuclear translocation of NF-κB (12, 19). Beyond NF-κB activation, NOD1 recruitment of RIP-2 is believed to activate c-jun kinase (JNK) (20, 21).

### Inflammasomes

NLRPs represent the largest NLR subfamily and are characterized by the presence of an N-terminal PYD effector domain (11, 22). Activation of various NLRPs by specific agonists leads to assembly of a multi-protein inflammasome complex. During inflammasome activation, the activated NLRP recruits the bipartite PYD-CARD domain protein ASC (also known as PyCARD) though a PYD–PYD interaction and the CARD domain of ASC subsequently recruits the CARD domain of caspase-1 (4) (Figure 1). In the assembled inflammasome, proximity-induced auto-activation of the catalytic domain of caspase-1 results in the mature, fully active caspase-1 followed by proteolytic cleavage and release of IL-1β and IL-18 (23, 24). Unlike NODosomes, the critical interaction to assemble NLRP inflammasomes is the PYD–PYD interaction with ASC. Two typical types of NLRP inflammasomes have been identified (25, 26). The NLRP1 inflammasome is composed of NLRP1, the adapter protein ASC, ASC-recruited caspase-1, and caspase-5 which is recruited through a NLRP1-specific C-terminal CARD domain (27). The NLRP3 inflammasome is believed to represent the arrangement of most NLRP inflammasomes and contains NLRP3, ASC, and caspase-1, but does not recruit caspase-5 (28). One other class of NLR-inflammasome is represented by NLRC4 (Ipaf/Naip5). By virtue of its N-terminal CARD domain, NLRC4 which is activated by flagellin, is believed to directly recruit caspase-1 through a CARD–CARD interaction, independent of ASC (29, 30) (Figure 1). Despite differences in the mode of caspase-1 recruitment, all of these inflammasomes control the processing and activation of the pro-inflammatory cytokines IL-1β and IL-18.

## Homo-Domain Interaction

Pyrin domains and CARD domains are members of the larger death domain (DD) fold superfamily characterized by the highly similar secondary structure of an antiparallel, six α-helical bundle (31). Like their close cousins, members of the DD and death effector domain (DED) family which interact homotypically (32), it is widely held that PYD- and CARD-containing proteins act exclusively in similar fashion.

### Pyrin domains

The PYD domain is a conserved sequence motif found in more than 20 human proteins with putative functions in apoptotic and inflammatory signaling pathways (33). Studies reporting PYD structures are rare, due to the limited solubility of these domains (31). However, several PYDs including those from ASC, POP1, and NLRP3 have been structurally characterized. All have distinct positively and negatively charged surface patches in their structure, leading to the proposal that electrostatic interactions are critical for PYD interaction (33–36). Notably, the three-dimensional structure of the human ASC PYD has helped clarify how this adaptor protein binds to the PYD of NLRP3 as well as that of POP1 (33, 37). The PYD domain of ASC is a highly bipolar molecule with most of the positively charged side-chains located in helices 2 and 3 and the connecting loop, while most of the negatively charged side-chains reside in helices 1 and 4 and the immediately adjacent regions, suggesting that, in analogy to CARD domains, charge-charge interactions may play an important role in PYD domain interactions (38). In fact, the negative residues on the ASC PYD Asp6, Asp10, Asp51, and Asp54 play important role in the interaction between ASC and POP1. Consistently, at least two positively charged amino acids in POP1, including Lys21 and Arg41, were required for this association (39).

### CARD domains

CARD domains are thought to homotypically interact in a fashion similar to PYDs. Structural studies of adaptor proteins such as RAIDD and the NLR-like apoptosome protein Apaf-1 revealed that CARD domains also contain distinct basic and acidic patches (38, 40–42), suggesting an electrostatic nature for CARD–CARD interaction. Indeed, the acidic surface of the Apaf-1 CARD located in helices 2 and 3 is necessary for interaction between Apaf-1 and caspase-9 (38, 42). In addition, hydrophobic interactions are also an important driving force underlying this particular CARD/CARD interaction (42).

## Regulation of NLR Assembly by POPs and COPs

Based on our current knowledge of NLR assembly, it is likely that one powerful way to modulate the assembly of NODosomes and inflammasomes is the disruption of PYD and CARD homo-domain interactions. The growing POPs and COPs families (as discussed in detail below) are potential modulators of inflammasome and NODosome assembly and are likely important to understanding NLR-associated diseases.

**Figure 2:**
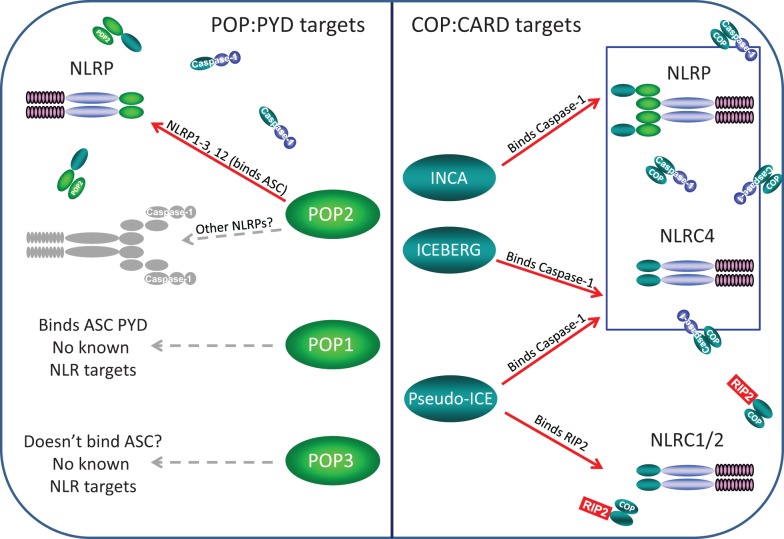
**Known and likely targets for human POPs and COPs**. Left panel, only POP2 is known to limit assembly of certain NLRP inflammasomes but whether POP2 restricts all NLRP inflammasomes is unknown. POP1 may be able to inhibit some NLRP:ASC interactions, but these remain unknown. While POP3 may likely be unable to bind ASC, whether it might interact with an NLRP PYDs remains to be determined. Right panel, INCA, ICEBERG, and COP/Pseudo-ICE all bind pro-Caspase-1 and should inhibit all NLRP and NLRC inflammasome, however, no specific inflammasome targets have been examined. However, only COP/Pseudo-ICE interacts specifically with the CARD of RIP-2, thus it may be a singular regulator of NODosomes.

Pyrin-only proteins and COPs are relatively short proteins of approximately 90 amino acids composed essentially of only a PYD or CARD domain. Accordingly, they are structurally and functionally related, and as expected, homotypic interactions appear key to their inhibitory effects in regulating NLR assembly. The currently known POPs and COPs and their known or likely roles in regulation of NLR complexes are depicted in Figure 2.

## Pyrin-only Proteins

To date, two POPs have been characterized based following initial identification in the human genome using bioinformatic mining. These include POP1 (*PYDC1*) and POP2 (*PYDC2*). An evolutionary analysis of POP2 suggests the possibility of a third POP within the NLRP2P pseudogene locus (43). Both appear to have resulted from either gene duplication (POP1) or retrogene insertion (POP2) from PYD-encoding genes already present in an ancestral primate genome (43, 44) and thus do not represent lateral gene transfer of viral PYD sequences. Both POPs exhibit NF-κB modulating activity, but contrary to numerous reviews which incorrectly ascribe inflammasome inhibitory properties to POP1, only POP2 is known to inhibit inflammasome assembly. Curiously, certain myxoma viruses belonging to the poxviridae employ POPs (vPOPs) as pathogenic determinants.

### POP1

In an early bioinformatics screen for Pyrin-containing sequences, Stehlik et al. discovered the first POP1, initially designated ASC2 due to the high degree of similarity with the ASC PYD domain (44, 45). The POP1 gene is located on chromosome 16p12.1 and consists of two exons interrupted by a 580 bp intron, where the entire PYD is encoded in a single exon yielding an 89 amino acid sequence which is 64% identical (88% similar) to the PYD domain of ASC (44). Expressed predominantly in monocytes, macrophages, and granulocytes, POP1 is implicated in regulating inflammation. POP1 inhibits NF-κB activation by a variety of stimuli, including TNFα, IL-1β, Nod1, and Bcl-10, in HEK-293, COS-7, and Hela cells (44). POP1 interacts with the PYD of ASC and also suppresses NF-κB activity stimulated by co-expression of ASC and pyrin or NLRP3. The capacity of co-transfected POP1 to inhibit NF-κB activation induced by various proteins including TRAF2, TRAF6, the TRAF-binding kinase TBK1, the IKK complex constituents IKKα and IKKβ, and by the IKK-related kinase IKKi, revealed that POP1 modulates NF-κB activation at the level of the IKK complex, as reported for ASC and for NALP4/NLRP4 (46, 47). Consistently, POP1 associates with the IκBα kinases IKKα and IKKβ, inhibiting their kinase activity. These associations are very unlikely to be the result of homotypic domain interactions as IKKs lack PYD. Instead, a more likely possibility is that POP1 contains small IKK-inhibiting sequence similar to the 23 amino acid NEMO binding domain (48). It is anticipated, although not yet demonstrated, that POP1 should inhibit IKK activity downstream of the NODosome activation of RIP-2. Although POP1 enhanced ASC-mediated IL-1β activation (44), in a later study, POP1 did not inhibit either ASC-dependent or NLRP3 inflammasome processing of IL-1β (49). Thus, no evidence to date supports a role for POP1 in inhibiting an inflammasome, consistent with a structure-predicted inability to regulate NLRP3 inflammasome activation (39). Taken together, it is likely that POP1 targets NF-κB activity via inhibition of IKK, but does not act as an inhibitor of ASC-dependent NLRP inflammasomes. Despite the strong molecular evidence for the function of POP1, there have been no studies of its function using primary human or POP1-expressing mouse myeloid cells.

### POP2

Following the discovery of POP1, a second human POP (POP2) was identified simultaneously by two independent groups (50, 51). POP2 is encoded by a 294 nucleotide, single exon gene located on human chromosome 3q28 and produces a protein of 97 amino acids with high similarly to the PYDs of NLRP2 and NLRP7 (78% similarity, 67% identity to the PYD of NLRP2) (50, 51). POP2 is expressed in human testis, primary peripheral blood leukocytes, monocytic cell lines, and is induced in human primary monocytes or monocytic cell lines by a variety of stimuli (PMA, LPS, or TNF-α) (49, 50), implicating a function in inflammation and host immunity. Despite the absence of a canonical nuclear localization signal sequence, POP2 displays both cytoplasmic and nuclear expression patterns in transfected cells, suggesting that POP2 may function in either, or both, compartments (50). As postulated from observations with POP1 and other PYD-containing proteins, POP2 diminishes NF-κB activation. As anticipated, POP2 blocks TNF-α-mediated NF-κB activation (50). However, POP2 inhibits the activity of transfected NF-κB p65/RelA, indicating that POP2 acts distally in the NF-κB cascade at the level of p65, a function that POP1 and other PYDs apparently lack. Recent work reveals that POP2 acts to limit the transactivation potential of the C-terminal transcriptional activation domain 1 (TAD1) of p65/RelA (49). How this is accomplished is unclear, but one possibility is a blockade of one or more of the kinases responsible for necessary phosphorylation events within TAD1 of p65 such that transactivation potential is reduced. In addition, although POP2 interacts with ASC, POP2 inhibition of NF-κB p65 is ASC-independent (49).

The interaction of POP2 with ASC leads to formation of a peri-nuclear “speck” structure similar to that observed with co-expression of ASC with Pyrin, POP1, various NLRPs, and upon NLRP3 inflammasome activation (50, 51). Moreover, POP2 also interacts with several PYD-containing NLRs (NLRP1, 2, 3, and 12) (50, 51). Indeed, POP2 nearly abolishes the interaction between ASC and the NLRP3-PYD as well as inhibiting ASC interaction with NLRP1 and NLRP12 although to a lesser extent (50). Thus, POP2 may be a broad acting competitive inhibitor of inflammasomes. This notion is supported by recent experiments revealing that POP2 effectively inhibits the non-NLR AIM2 inflammasome (unpublished observation). Co-expression of POP2 with ASC and the disease-associated NLRP3 R260W mutant impairs activation of caspase-1 in a dose-dependent manner (51). NLRP3 R260W (like other disease producing NLRP3 variants) interacts more readily/efficiently with ASC leading to caspase-1 activation, even without an agonist (51), suggesting that POP2 limits on NLRP3 inflammasome activity may not be overcome by activating mutations. POP2 also impairs activation of the NLRP2 inflammasome (51). These *in vitro* observations demonstrate the broad potential of POP2 to disrupt NLRP-ASC interactions and have significant implications for the *in vivo* role of POP2.

By generating POP2 truncation mutants with inter-helical stop codons to maintain the integrity of the remaining helices, Atianand et al. probed the specific portions of POP2 required for its function. In this study, the first α-helix of POP2 (residues 1–19) was shown to be both necessary and sufficient for inhibiting transactivation by NF-κB and for restricting inflammasome assembly (49). The first α-helix of POP2 has no basic residues but contains three acidic residues Glu^6^, Asp^8^, Glu^16^ (49). Consistent with the structural data showing the importance of electrostatic surface patches (EPSPs) in PYD–PYD interactions (39) mutation of these acidic residues markedly impairs the ability of POP2 to disrupt inflammasome function. NF-κB inhibition is seemingly unaltered by these mutations, implicating other elements of the first helix which might reside on the opposite helical face, although this remains to be established. The ASC adaptor protein has a bipartite charge distribution, with both positive and negative EPSPs (39). Although the negative EPSP of ASC was demonstrated to be a POP1 binding site, the presence of necessary acidic residues in the first α-helix of POP2 (and another contributing acidic residue in helix 4), leads to the proposal that helices 1 and 4 of POP2 interact with the positive EPSP on helix 2 and 3 of the ASC PYD. Interestingly, the positive ASC helix 2/3 EPSP was also proposed as the binding site of NLRP3 (39), suggesting that POP2 inhibits NLRP3 inflammasome assembly through competition with NLRP3 for this site on ASC.

### Other human POPs

Our evolutionary analysis of POP2 suggests the possibility of a third POP (POP3) within the human genome, encoded by an open reading frame within the NLRP2P pseudogene (43). Based on sequence similarity, a protein produced by this ORF would be anticipated to possess the NF-κB inhibitory properties of POP2, but lack the ability to inhibit the NLRP3 inflammasome as the acidic residues known to be important for inhibition are non-charged, non-polar. Unfortunately, the details of this additional potential POP protein encoding gene await an initial description.

### vPOPs

Some viral proteins, such as Myxoma virus protein M013 and Shope fibroma virus protein (SFV-gp013L) have also been described to inhibit PYD-dependent inflammasomes and impair NF-κB activity (52–54). Johnston et al. identified the M13L gene which encodes the PYD-containing protein M013 in Myxoma virus, a rabbit-specific poxvirus that is the causative agent of the lethal disease myxomatosis (52). Notably, in the absence of the M013 vPOP, rabbits readily clear the viral infection and survive, indicating the key role of M013 in pathogenesis. Interestingly, genes encoding additional viral POPs closely related to M13L were found in other poxviruses, including Yaba-like disease virus, Tanapox virus, Shope fibroma virus (gene S013L, protein gp013), Mule deer poxvirus, and Swinepox virus, suggesting the conservation of PYD proteins which likely benefit viral replication and virulence among diverse poxvirus genera (52, 53, 55). As with human POPs, the vPOPs M013 and gp013L interact directly with ASC through PYD–PYD interaction, thus preventing activation of NLRP3, consequently reducing protective bioactive IL-1β, IL-18 production (52, 53). Interestingly, the first 22 amino acids of M013 vPOP were required for ASC binding, formation of peri-nuclear specks, and subsequent inhibition of caspase-1 activation as seen with the first α-helix (19 residues) of human POP2 (49, 52). Both M013 and SFV-gp013L vPOPs impact NF-κB activity. While the SFV-gp013L vPOP enhances activation of NF-κB independently of ASC and NLRs (53), M013 vPOP inhibits NF-κB, binds directly to host NF-κB1, inhibits the translocation of p65 to the nucleus (54), reminiscent of human POP2 (50). Interaction between M013 vPOP and NF-κB1 may interfere with NF-κB1/p105 degradation thus preventing p50 release, formation and subsequent nuclear translocation of the active p50/p65 heterodimer (54). Accordingly, infection of THP-1 cells with M013-deficient myxoma virus led to rapid secretion of cytokines such as TNFα, IL-6, and MCP-1 (54). While the competitive binding of M013 vPOP to ASC is required for the disruption of NLRP3 inflammasome, as with POP2 this interaction was dispensable for NF-κB inhibition (56). The sequence and functional similarity between the first helices of POP2 and M013 suggests the use of similar dual mechanisms to provide the presumed beneficial control of inflammation by POP2 but prevent protective anti-viral inflammatory responses through M013.

### Summary

Among the identified human POPs, POP2 is the only one with a demonstrated capacity to restrain assembly and function of NLR complexes, although both POP1 and POP2 impact NF-κB signaling. Two distinct interaction surfaces on the first α-helix of POP2 likely account for its unique ability to both limit NF-κB activation and restrict inflammasome formation. Thus, POP2 is likely a multifunctional and broadly acting regulator of inflammatory signal pathways in higher primates, while in contrast, POP1 inhibits upstream aspects of NF-κB signaling. The biological role of these POPs, although intriguing and of highly probable significance, remains less clear. Similar to human POP2, the vPOP M013 also has a dual role in impairing NF-κB activation and inflammasome formation, representing a viral strategy to circumvent protective innate inflammatory responses. Future investigation is needed to clarify the mechanism(s) by which human as well as viral POPs modulate NF-κB activity.

## CARD-only Proteins

The human genome also contains three COPs, COP/Pseudo-ICE (*CARD16*), INCA (*CARD17*), and ICEBERG (*CARD18*) which most likely arose through gene duplication and as with the POPs are restricted to higher primates (57). These proteins were initially identified and described due to sequence similarity with caspase-1 and serve as non-enzymatic decoys regulating caspase-1 activity. To date, there are no clear demonstrations of viral COPs.

### COP/Pseudo-ICE

The 16th human CARD (CARD 16) was identified as a 97 amino acid protein consisting of a CARD region (residues 1–91) with 97% identity to the CARD of caspase-1 (58). This protein is encoded on chromosome 11p22, in the same region where caspase-1, caspase-4, caspase-5, and ICEBERG (see below) reside, and is composed of three exons separated by introns of 631 and 844 bp respectively, such that the majority of the coding sequence and the entire CARD domain derive from exon 2 (57–59). Since CARD16 contains essentially only a CARD domain, this protein was named COP or Pseudo-ICE due to its high sequence identity with the CARD domain of caspase-1 [also known as Interleukin-1β converting enzyme (ICE)] (59). To avoid confusion between COP and the generic group of COP proteins, we will refer to this protein as COP/Pseudo-ICE. COP/Pseudo-ICE is expressed mainly in placenta, spleen, lymph node, and bone marrow and in the THP-1 cell line (58, 59). Binding caspase-1, RIP-2, and self-associating through its CARD domain, COP/Pseudo-ICE inhibits RIP-2-mediated oligomerization of caspase-1, thus blocking the activation of caspase-1 and subsequent release of mature IL-1β (58, 59). Overexpression of COP/Pseudo-ICE can trigger NF-κB induction and enhanced TNF-α-induced NF-κB activation via a mechanism dependent on the IKK complex (59).

### INCA

The newest indentified COP named INCA (Inhibitory CARD/CARD17) is located on human chromosome 11q22, between COP/Pseudo-ICE and ICEBERG (57, 60). The predicted INCA cDNA sequence is composed of four exons and three introns 628, 1092, and 6778 bp in length (60). The open reading frame spans the first to the third exon, but as with COP/Pseduo-ICE, exon 2 encodes most of the open reading frame including the CARD domain. Exon 4 is non-coding and thus functions as a 3′-untranslated region (60). Like COP/Pseudo-ICE and ICEBERG, although INCA contains 110 amino acids, the essential CARD domain consists of the first 91 residues and shares 81% sequence identity with the CARD of caspase-1. INCA is expressed in a wide variety of human tissues with highest expression in brain, heart, spleen, lung, and salivary gland. In general, in tissues expressing INCA, caspase-1 is also expressed, with the exception of the salivary gland. Interestingly, INCA and caspase-1 are coordinately upregulated upon stimulation with INF-γ in the monocytic cell lines THP-1 and U937, however, LPS and TNF-α which also induce caspase-1, fail to upregulate INCA (60). INCA does not interact with the NF-κB-activating kinase RIP-2, but like COP/Pseudo-ICE, INCA can self-associate, bind to the pro-domain of caspase-1, and cross-associate with the other COPs (60). Like ICEBERG which also fails to bind RIP-2, INCA was completely incapable of activating NF-κB itself and does not inhibit NF-κB activation induced by well-known factors such as TNF, caspase-1, COP/Pseudo-ICE, or RIP-2. However, INCA significantly reduces the release of mature IL-1β from THP-1 cells (comparable to COP/Pseudo-ICE), probably due to its interaction with caspase-1 (60).

### ICEBERG

EST clone AA046000 contains a 273 bp open reading frame that codes for a 90-residue protein named ICEBERG (59) which is 53% identical to the CARD domain of human caspase-1 (61). ICEBERG is detected mainly in placenta and in many human cell lines and its expression is upregulated by LPS and TNF in THP-1 monocytes (61). ICEBERG can self-associate, bind to the pro-domain of caspase-1, and cross-associate with another COP/Pseudo-ICE through its CARD domain. Although unable to interact with RIP-2, ICEBERG clearly binds to caspase-1 through charge–charge interaction between their CARD domains; not surprisingly ICEBERG is unable to activate NF-κB (59, 61). However, RIP-2 independently binds and activates caspase-1 directly via CARD–CARD interaction (61, 62) and ICEBERG blocks this interaction by binding caspase-1, a competitive inhibition that disrupt oligomerization of RIP-2 and caspase-1 and consequently inhibits IL-1β production.

### Summary

All three human COPs interact with caspase-1 and thus, when expressed are anticipated to influence all instances of inflammasome-elicited IL-1β and IL-18 production. Only COP/Pseudo-ICE targets events mediated through the CARD domain of RIP-2. Why three independent regulators are required and how they differ is largely unknown and unstudied, but their common expression in the placenta and monocytic cells may suggest roles in development as well as immunity.

## Evolutionary History of POPs/COPs

In the battle between pathogens and host immunity, pathogens, typically viruses, manifest sophisticated mechanisms to escape the detection and control of host immune systems. In contrast, the host defense incessantly develops strategies to eliminate pathogens without becoming hyper-responsive and causing harm to itself. Evolution plays a pivotal role in these both processes. Poxviruses are excellent examples for studying genome evolution. They accumulate point mutations at relatively low rates, whereas gene duplications, losses, gain by horizontal gene transfer, and recombination between different viral species occur frequently (55). These events led to the appearance of several groups of genetic elements known as host range genes thought to be important for host adaptation and subverting the host anti-viral response [listed in (55)]. Of these host range genes, M013 vPOP protein was proposed to be an evolutionary factor based on its high sequence similarity with pyrin, particularly within the first 50 residues and its role as a competitive inhibitor of ASC interactions (52, 53). It is possible that M013 vPOP is the product of ancestral capture, recombination, and re-assortment events that occurred during co-evolution of the virus and its host. Interestingly, phylogenetic analyses showed that, together with Myxoma virus, other poxviruses belonging to the largest subgrouping of Chord poxviruses possess M013 orthologs. These include Yaba-like disease virus, Tanapox virus, Shope fibroma virus, Mule deer poxvirus, and Swinepox virus (55), suggesting a shared evolutionary ancestor that may have acquired a host PYD domain prior to the divergence of this group of viruses.

**Figure 3:**
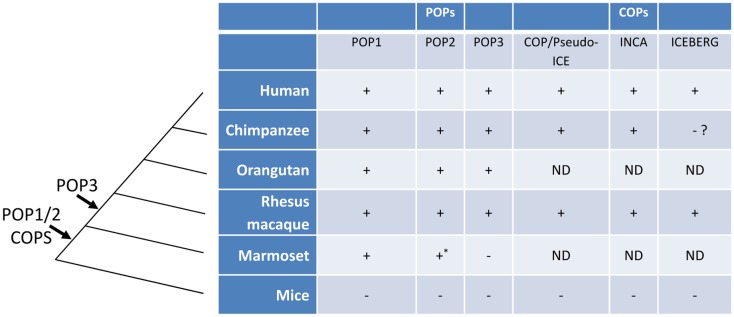
**The evolution of POPs and COPs**. POPs and COPs emerged very recently during mammalian evolution and are restricted to primates. All currently known POPs are present as intact coding regions in the complete genomes of Old World primates and hominids but are absent from the complete genomes of mice, non-primates, and, for at least POP2 and POP3, New World primates. All currently known COPs are primate restricted [an ICEBERG locus has been reported for the treeshrew a primate ancestor, see (63)]. Arrows indicate the relative timing of the retro-transposon insertion of POP2/3 and the duplication events leading to POP1/COPs in an ancestral primate genome. (ND, not determined; *, non-functional).

Among mammals, the human genome encodes at least two and possibly three POPs (POP1, POP2, and the putative POP3) and syntenic orthologs are present in the genomes of the non-human primates *Pan troglodytes* (chimpanzee), *Macaca mulatta* (rhesus macaque), *Pongo abelii* (orangutan) but the NLRP2P locus (POP3) and a functional POP2 appear to be absent in that of marmosets (43). Interestingly, POPs were not found in the genomes of mice, rats, and domestic animals, even though most human NLRPs are similar to those in other species (11, 43). Taken together, these observations support the current hypothesis that POPs are evolutionary recent developments in the mammalian genome and may be limited to hominids and Old World primates. The evolutionary history of POPs and COPs are summarized in Figure 3.

Although the selective pressures driving the appearance of human POPs are unknown, their origins and evolutionary pathways have been investigated. The POP1 gene is located on chromosome 16p12.1, the same chromosomal band as the ASC gene, approximately 14 kb away and the PYD of POP1 is most closely related to that of ASC (88% similarity and 64% identity) (44). This close proximity as well as the strong sequence similarity suggests that POP1 arose by gene duplication (44). More interestingly, phylogenetic analysis revealed that both POP2 and NLRP2P arose from the same ancestor, an NLRP2/7-like gene (designated Nlrp2 in mouse), most likely through retrogene insertion events during the course of primate evolution resulting in two distinct, and seemingly non-functional, pseudogenes (43). Curiously, during higher primate evolution, POP2 gained a functional promoter, lost the remnants of the non-PYD portions of the ancestral NLRP2/7-like gene, and acquired a new polyadenylation sites (43). Despite the sequence similarity, NLRP2 is adjacent to NLRP7 on chromosome 19 in human and chimpanzee genomes, whereas POP2 and NLRP2P are located on chromosome 3 and chromosome X, respectively. The distal location and the absence of introns as well as the presence of stop-codon disrupted NBD and LRR sequences in the founding primate genomes strongly implicates the retro-transposition of NLRP2/7-like mRNAs yielding processed pseudogenes.

Chimpanzee POP2 is identical to human POP2 at both the DNA and protein level (43). Macaque POP2 is highly conserved, but not identical to the human sequence. Although macaque POP2 retains both functions of the human protein, it is a less robust inhibitor which may result from an additional C-terminal 41 amino acid stretch absent from human and chimpanzee POP2 (43). Among the New World primates, the marmoset genome contains an intron-less pseudogene sequence without a start codon with remnants of not only the PYD, but the nucleotide-binding domain and portions of the LRR sequences, which share identity with NLRP2/7. Under strong selective pressure during divergence of New World and Old World primates, the NBD and LRR sequences were lost to produce a functional POP2, retained in the common ancestor of macaques and higher primates. Further pressure during the emergence of hominids purified the developing POP2 PYD sequence to yield a version of POP2 with stronger inhibitory activities on NF-κB and inflammasomes which is invariant between humans and chimpanzees (43).

The other NLRP2/7-derived pseudogene named CLRX.1/NOD24 (NLRP2P) also contains an open reading frame that may encode POP3, but unlike POP2 it contains a conserved stop codon that would preclude PYD helices 4 through 6.

Similar to POPs, the three mammalian COPs (COP, INCA, ICEBERG) are also found only in humans and primates (63). No ortholog has been identified in the same locus in mouse and rat genome (57). All three COPs are highly homologous with the CARD domain of caspase-1 and are located on the same chromosome adjacent to caspase-1 (58, 60, 61), suggesting that COPs emerged by gene duplication. While putative orthologs of COP and INCA were identified in human, chimpanzee, and Rhesus macaque, ICEBERG was only found in human and Rhesus monkeys despite the earlier divergence of these monkeys from the human-chimpanzee lineage (57), perhaps owing to the incompleteness of the chimpanzee genome at this locus on chromosome 9. Nevertheless, like POPs, COPs appear restricted to hominids and primates, suggesting that strong selective pressures, perhaps acting to control inflammation at both level of cytokine gene transcription and processing of IL-1β by inflammasome activated caspase-1, drove the emergence of at least six independent genes with largely unexplored and unexplained biological roles.

## Biological Impact

Given the key role of IL-1β in inflammation, it is not surprising that defective control of the inflammasome appears to be a feature of many inflammatory diseases. In fact, the initial description of the NLRP3 inflammasome and clinical interest in defective control of inflammasome activation resulted in large part from genetic analysis of families with autoinflammatory diseases. These include familial Mediterranean fever (FMF), Muckle-Wells syndrome (MWS), familial cold autoinflammatory syndrome (FCAS), neonatal-onset multisystem inflammatory disease/chronic infantile neurological cutaneous, and articular syndrome (NOMID/CINCA) (64–67). Of these, FMF is the first known disease with associated mutations in the Pyrin protein, a small non-NLR protein composed of an N-terminal PYD domain and a C-terminal PRY-SPRY domain (68). Like NLRPs, Pyrin associates with ASC and may form an inflammasome (69, 70), however, most disease-associated Pyrin mutations occur in the PRY-SPRY domain which is functionally uncharacterized. Unexpectedly, the other hereditary fever syndromes, including FCAS, MWS, CINCA, which range from the very mild FCAS to the severe and chronic NOMID/CINCA, are all attributable to NLRP3 (CIAS1/Cryopyrin). Even though being distinct clinical entities, the majority of disease-associated mutations are located in exon 3 which encodes the NACHT (NBD/NOD) domain (66, 67). It is likely that these mutations cause spontaneous oligomerization of the NACHT domain due to decreased interaction with the LRR which are thought to inhibit auto-activation of NLR-inflammasomes (10) which in turn results in overproduction of IL-1β and IL-18, leading to chronic, excessive inflammation. Other inflammation-related diseases have been reported. Blau syndrome and Crohn’s disease are associated with the CARD-containing NLR, NOD2/NLRC2 with NACHT domain mutations occurring in Blau syndrome (71), while LRR domain mutations of NOD2 are reported for Crohn’s disease (13, 72). Moreover, through rapidly growing clinical and NLR-deficient mouse studies, specific NLRs have been linked to numerous diseases, including NLRP1 [various autoimmune diseases (e.g., psoriasis, systemic lupus erythematosus, rheumatoid arthritis, Celiac disease, and type 1 diabetes), NLRP3 (e.g., gout, atherosclerosis, type II diabetes, rheumatoid arthritis, Alzheimer’s, and cancer of the colon and skin), NLRP6 (e.g., fatty liver disease, inflammatory bowel disease, and gastric cancer), NLRP12 (atopic dermatitis), and NLRC4 (e.g., gastric cancer and inflammatory bowel disease); reviewed in (5, 73)]. The non-NLR PYD-containing protein AIM2 also forms an inflammasome and is implicated in allergy [reviewed in (5)]. The importance of inflammasomes in complex diseases, the majority of which have underlying inflammatory etiologies, underscores the need for tight control of inflammasome activity as well as other inflammatory signaling pathways to maintain homeostasis.

In humans, POPs and COPs very likely represent a mechanism for restraining pro-inflammatory activation events, for terminating inflammasome signals, or both depending on the specific protein. Given that POP2 disrupts downstream activation of the NLRP3 and likely other NLR-inflammasomes and concomitant release of IL-1β, POP2 may be critically important in establishing the normal limits of inflammasome activation. This has significant implications for inflammasome-associated diseases. For example, mutations in POP2 involving the acidic amino acids of the first α helix that are important for NLRP3 inflammasome inhibition may result in diseases similar to those seen with NLRP3 mutations. Likewise, mutations that diminish expression of POP2 may have broad effects allowing more ready and sustained activation of various NLRPs with attendant increased pathologic inflammation. Gain of function mutations might provide a protective benefit for NLRP-associated diseases, although diminished inflammatory responses might similarly predispose individuals to infection or interfere with wound healing. Similarly, COPs directly binds caspase-1 and therefore interfere with the interaction of caspase-1 with ASC (in inflammasomes) and/or with RIP-2 (in NODsomes). Consequently, COPs potentially target NLRCs such as NOD1, NOD2, and NLRC4 as well as NLRPs, and in principle any pathway requiring a CARD:CARD domain interaction. The combined regulatory potential of POPs and COPs within NLR biology is thus staggering. Further, the evolutionary evidence suggests that it is critical for human survival. Despite the implications, little attention has been devoted to understanding these small, seemingly insignificant proteins.

## Outstanding Questions

In light of the profound biological consequences of NLR auto-activation (chronic inflammation), tight control of NLR protein functions is critical to provide the delicate balance between the initiation and perpetuation of immune responses and anti-inflammation mechanisms. POPs and COPs are preeminent candidates as they are able to control of both NF-κB and inflammasome activation. However, studies of the mechanism(s) by which they interfere with these processes are few. Whether POPs and COPs represent an array of modulators with overlapping functions or are discrete, sufficient, regulators of different aspects of NF-κB signaling, and/or inflammasome activity is unknown. Several outstanding questions are of immediate importance to the field. Beyond more immediate questions such as which tissues and cells express POPs and COPs and under what conditions, the question of what roles POPs and COPs play in normal human physiology and pathology is the most urgent. Mouse models of POP/COP *in vivo* function need to be developed to study these primate-restricted proteins. Gene association/single nucleotide polymorphism studies of human inflammation-related disease without specific NLR mutations are also likely informative. Whether POPs and COPs are broadly acting regulators or specific to particular pathways remains unclear, but the answer certainly resides at the intersection of mechanistic studies aimed at understanding the distinctions between family members and the insights provided from *in vivo* systems. More broadly, the presence of POPs and COPs in humans implies fundamental differences in some, if not many, aspects of NLR biology between mice and humans that at present remain mysteries. Finally, lessons learned from the study of POPs and COPs may lead to additional biologics that can specifically target dysregulated inflammatory processes mediated through PYD and CARD domain-containing protein complexes.

## Conflict of Interest Statement

The authors declare that the research was conducted in the absence of any commercial or financial relationships that could be construed as a potential conflict of interest.
